# Endocardial to Myocardial Notch-Wnt-Bmp Axis Regulates Early Heart Valve Development

**DOI:** 10.1371/journal.pone.0060244

**Published:** 2013-04-01

**Authors:** Yidong Wang, Bingruo Wu, Alyssa A. Chamberlain, Wendy Lui, Pratistha Koirala, Katalin Susztak, Diana Klein, Verdon Taylor, Bin Zhou

**Affiliations:** 1 State Key Laboratory of Biotherapy and Cancer Center, West China Hospital, West China Medical School, Sichuan University, Chengdu, Sichuan, China; 2 Department of Genetics, Albert Einstein College of Medicine, Bronx, New York, United States of America; 3 Renal, Electrolyte and Hypertension Division, University of Pennsylvania, Philadelphia, Pennsylvania, United States of America; 4 Institute of Anatomy, University Hospital Essen, Essen, North Rhine-Westphalia, Germany; 5 Department of Biomedicine, University of Basel, Basel, Switzerland; 6 Departments of Pediatrics and Medicine (Cardiology), The Wilf Family Cardiovascular Research Institute, Albert Einstein College of Medicine, Bronx, New York, United States of America; 7 Department of Cardiology, The First Affiliated Hospital of Nanjing Medical University and Jiangsu Province Hospital, Nanjing, Jiangsu, China; National Cancer Center, Japan

## Abstract

Endocardial to mesenchymal transformation (EMT) is a fundamental cellular process required for heart valve formation. Notch, Wnt and Bmp pathways are known to regulate this process. To further address how these pathways coordinate in the process, we specifically disrupted *Notch1* or *Jagged1* in the endocardium of mouse embryonic hearts and showed that Jagged1-Notch1 signaling in the endocardium is essential for EMT and early valvular cushion formation. qPCR and RNA *in situ* hybridization assays reveal that endocardial Jagged1-Notch1 signaling regulates *Wnt4* expression in the atrioventricular canal (AVC) endocardium and *Bmp2* in the AVC myocardium. Whole embryo cultures treated with Wnt4 or Wnt inhibitory factor 1 (Wif1) show that *Bmp2* expression in the AVC myocardium is dependent on Wnt activity; Wnt4 also reinstates *Bmp2* expression in the AVC myocardium of endocardial *Notch1* null embryos. Furthermore, while both Wnt4 and Bmp2 rescue the defective EMT resulting from Notch inhibition, Wnt4 requires Bmp for its action. These results demonstrate that Jagged1-Notch1 signaling in endocardial cells induces the expression of Wnt4, which subsequently acts as a paracrine factor to upregulate Bmp2 expression in the adjacent AVC myocardium to signal EMT.

## Introduction

Formation of heart valves is critical for heart function and is required for both embryogenesis and postnatal life. Defects in this process may cause congenital heart valve disease [1], [2], [3]. In mice, heart valve development begins with the mesenchymal transformation of endocardial cells (EMT) at embryonic day (E) 9.5 and E10.5 at endocardial cushions of atrioventricular canal (AVC) and outflow tract (OFT). EMT consists of a multiple cellular events including delamination of endocardial cells from the AVC endocardium, their invasion into the extracellular matrix, and the acquisition of mesenchymal phenotypes [4], [5], [6], [7], [8], [9]. Even during EMT, endocardial cushions provide a valve-like function to prevent blood regurgitation in the developing heart [10].

Studies of EMT have identified Notch, Wnt, and Bmp pathways that act in the endocardium and/or myocardium of cushions to regulate this process. Global loss of Notch1 [11] or pan-endothelial loss of its ligand, Jagged1 [12] in the Notch pathway causes defective EMT, leading to hypocellular endocardial cushions. Similarly, ablation of endothelial Wnt [13] and myocardial Bmp [14], [15] activities inhibit EMT and valve development. Furthermore, Ma and Martin *et al.* have reported that myocardial *Bmp2* is required for endocardial *Notch1* expression [15], and studies by Luna-Zurita and De la Pompa *et al.* have shown that Bmp2 drives EMT of ventricular endocardial cells which ectopically express active Notch1 [16]. How Notch, Wnt, and Bmp pathways coordinate in EMT process is still incompletely understood.

Global or pan-endothelial disruption of the Notch pathway also results in early vascular defects [17], [18], [19], in addition to the cardiac defects aforesaid. In this study, we seek to define specifically the role of endocardially produced Notch1 and Jagged1 in heart development by endocardial-specific deletion of *Notch1* or *Jagged1*. We show that endocardial Jagged1-Notch1 signaling is required for EMT and regulates *Wnt4* expression in the endocardium and *Bmp2* expression in the myocardium. We also reveal that Wnt4 regulates *Bmp2* expression. We further show that either Wnt4 or Bmp2 treatment rescues defective EMT resulting from Notch inhibition and Wnt4 rescuing requires Bmp activities. These results thus establish an endocardial to myocardial Notch-Wnt-Bmp signaling cascade essential for EMT during heart valve development.

## Methods

### Mice

The endocardial Cre mouse line (*Nfatc1^Cre^*) has an *IRES-Cre* cassette inserted at the 3′ untranslated region of the mouse *Nfatc1* [20] [21]. The lacZ *ROSA26* Cre reporter strain (*R26^fslz^*) [22] and *Notch1^f/f^* (*N1^f/f^*) [23] were obtained from the Jackson Laboratory (Bar Harbor, Maine). The *Jagged1^f/f^* (*J1^f/f^*) allele has been described previously [24]. The GFP Cre reporter line *R26^fsGFP^* [25], [26] was a gift from Gordon Fishell (New York University Medical School). All mouse strains were maintained on C57B6 background and mouse experiments were performed according to the guideline of the National Institute of Health and the protocol approved by the Institutional Animal Care and Use Committee of Albert Einstein College of Medicine (IACUC Approval Number: 20110303). Noontime on the day of detecting vaginal plugs was designated as E0.5. Mouse lines were PCR genotyped using tail or yolk sac DNA.

### Histology and Immunohistochemistry

E9.5 and E10.5 embryos were dissected and fixed overnight at 4°C using 4% paraformaldehyde (PFA) in phosphate-buffered saline (PBS). They were then dehydrated through an ethanol gradient, treated with xylene, and embedded in paraffin wax. Embryos were oriented for sagittal sections (6 µm) using a Leica microtome. Hematoxylin and eosin (H&E) staining was performed for histology. H&E stained tissue sections were examined and photographed using a Zeiss Axio Observer Z1 inverted microscope. For quantification of the number of cushion mesenchymal cells, serial sections across the cushion region were used for cell counting. The data were presented as the average number of cells per section from multiple embryos for each genotype. For immunochemistry, tissue sections were antigen retrieved by boiling for 10 minutes in sodium citrate (10 mM, pH 6.0) (Vector Laboratories) and blocked with 3% BSA in PBS before being incubated with primary antibodies and then secondary antibodies. Mouse anti-Notch1 antibodies (A6, Abcam) were used for detecting the membrane Notch1 as previously described [27]. The signal was amplified using the TSA Plus Cyanine 3 System (Perkin Elmer). Goat anti-jagged1 IgGs (C20, Santa Cruz) were used for detecting the membrane Jagged1. Immunostained tissue sections were photographed using a Leica TCS SP5 confocal scope.

### Cell Proliferation and Apoptosis Assays

Cell proliferation was determined by bromodeoxyuridine (BrdU) incorporation. A dose of 100 mg/kg BrdU (Invitrogen) was injected into the peritoneum of pregnant female mice two hours before isolation of embryos. E10.5 embryos were collected and fixed in 4% PFA at 4°C overnight. The fixed embryos were rinsed in PBS, dehydrated in a series of graded ethanol, paraffin embedded, and sectioned at 6 µm. BrdU-incorporated nuclei were detected using BrdU staining Kit (Invitrogen) following the manufacturer’s instructions. Serial sections of each embryo were examined and the results were presented as the mean percentage of BrdU-positive cells/total cells. The results from three age-matched control and endocardial *Notch1* null littermates were collected for statistical analysis. Cell apoptosis was analyzed by immunostaining using anti-cleaved-Caspase3 antibody (Cell Signaling Technology).

### RNA Extraction and Quantitative PCR (qPCR)

Total RNAs were isolated from pooled AVC tissues from five E10.5 hearts using Trizol (Invitrogen). First strand cDNA was synthesized using the Superscript II Reverse Transcriptase Kit (Invitrogen). qPCR was performed using Power SYBR Green PCR Master Mix (ABI). Gene specific primers were used (Table S1). The relative level of gene expression was normalized to an internal control (level of Gapdh) and calculated using the 2^-ΔΔCT^ method. Biological repeats were performed using three different samples for each genotype, and technical triplicates were carried out for each gene expression analysis. The mean relative expression of each gene between groups was used for statistical significant analysis.

### Wholemount X-gal Staining

Wholemount X-gal staining was performed as previously described [28]. E10.5 embryos were dissected, fixed in 4% PFA for 30 minutes at 4°C, washed twice in PBS containing 2 mM MgCl_2_ and once in PBS (pH 7.5)/2 mM MgCl_2_/0.1% deoxycholic acid/0.2% NP-40. The X-gal staining/reaction was developed in the same buffer containing 5 mM K3Fe(CN)6, 5 mM K4Fe(CN)6, and 0.6 mg/mL X-gal (Promega) at room temperature (RT) for six hours. The reaction was stopped by washing the embryos in PBS/0.5 mM EDTA. The stained embryos were photographed using a Zeiss Discovery V12 stereoscope. The stained embryos were then post-fixed, dehydrated, embedded in paraffin, sectioned and photographed using the Zeiss Axio Observer Z1 inverted microscope.

### Wholemount Pecam1 Antibody Staining

E10.5 embryos were fixed in 4% PFA in PBS for 2 hours at 4°C, dehydrated through a methanol series, and bleached in 6% H_2_O_2_/methanol for 1 hour. Embryos were then rehydrated, post-fixed in 4% PFA/0.2% glutaraldehyde for 20 minutes at room temperature and washed twice in PBST containing 0.1% Tween-20. The embryos were then blocked in PBSST containing 1% BSA and 0.1% Tween-20 for two hours, and incubated with Rat anti-Pecam1 antibodies (MEC13.3, BD Bioscience) in PBSST overnight at 4°C. The following day, the embryos were washed five times in PBSST and incubated overnight at 4°C with an HRP-conjugated donkey anti-rat secondary antibody (Vector Laboratories). On the third day, the embryos were washed five times in PBSST and incubated in A&B solution (Vector Laboratories) overnight at 4°C, before being washed four times in PBSST, rinsed once in PBST (PBS containing 0.1% tween20), and developed with the DAB Kit (Vector Laboratories). The stained embryos were then washed in PBS, post-fixed in 4% PFA, dehydrated through a methanol series, and imaged using a Zeiss discovery microscope. Three age-matched embryos from control or null groups were examined simultaneously.

### RNA *in situ* Hybridization

RNA *in situ* hybridization (ISH) was performed according to a previously described protocol [29]. Digoxigenin-labeled complementary RNA probes for *Wnt4* and *Bmp2* mRNA were prepared from the linearized plasmids by reverse transcription. E10.5 embryos were isolated in RNase-free PBS, fixed overnight in 4%PFA in PBS at 4°C, dehydrated through a methanol series, rehydrated and treated for one hour at room temperature (RT) with 3% H_2_O_2_ to quench the endogenous peroxidases. The embryos were then proteinase K (10 µg/mL) digested for 15 minutes at RT, refixed with 4% PFA/0.2% glutaraldehyde, and hybridized overnight at 70°C with antisense DIG-labeled RNA probes. The following day, the embryos were washed, blocked, and incubated overnight with an alkaline-phosphatase (AP) conjugate anti-DIG antibody. AP activity was detected using BM Purple (Roche). Embryos were post-fixed in 4%PFA/0.1% glutaraldehyde prior to visualization and imaging in PBS using a Zeiss discovery microscope. The stained embryos were next post-fixed, dehydrated, embedded in paraffin, sectioned and photographed using the Zeiss Axio Observer Z1 inverted microscope. For each gene, four to five age-matched embryos from control or mutant groups were analyzed simultaneously.

### Wnt2 Conditioned Medium

Wnt2-conditioned medium and control medium were prepared as described previously [30]. CHO cells were stably transfected with pcDNA3-mWnt2 or pcDNA3 vector. Cell lines were cultured in DMEM with 10% FBS until confluence. Medium was replaced with DMEM containing 0.5% FBS and the supernatant was collected after 24-hour culture and stored in −20°C.

### *In vitro* EMT Assay

EMT assay was performed as described previously [5], [9]. The AVC tissues were microdissected out from E9.5 hearts and cultured on rat-tail collagen gel in 4-well plates. Explants were cultured for 48 hours, and cells that migrated away from explants and invaded the gel were counted. For rescue experiments, DAPT (50 µM) (Tocris Bioscience), BMP2 (200 ng/ml) (R&D systems), Wnt2 conditioned medium, Wnt4 (250 µg/ml) (R&D systems), and Noggin (200 ng/ml) (R&D systems) were added to explant culture and changed after 24 hours.

### Whole Embryo Culture

Whole embryo culture was performed as described previously [31]. E9.5 embryos were dissected out in pre-warmed Hank’s Balanced Salt Solution (HBSS) (Sigma) with intact yolk sac. The embryos were cultured with whole embryo culture media containing 75% Rat whole embryo culture serum (Harlan, Laboratories, BT4520), 25% HBSS, 1% Pen-strep and 2 mg/mL of glucose. The embryos were cultured in incubator with specialized gas (60% oxygen, 5% CO_2_ and 35% N_2_) at 37 C for 24 hours. In some experiments, Wnt inhibitor Wif1 (2.5 µg/ml) (R&D systems) or Wnt4 (250 ng/ml) was added into the culture medium. The cultured embryos were used for gene expression assays using qPCR and/or RNA *in situ* Hybridization as described above.

### Statistical Analysis

Statistical analysis was performed using Microsoft Excel and all data were presented as mean ± SD. Student’s *t*-Test was used for comparison between groups and Probability (*p*) values <0.05 were considered as significant.

## Results

### Loss of *Notch1* or *Jagged1* in the Endocardium is Embryonic Lethal

**Figure 1:**
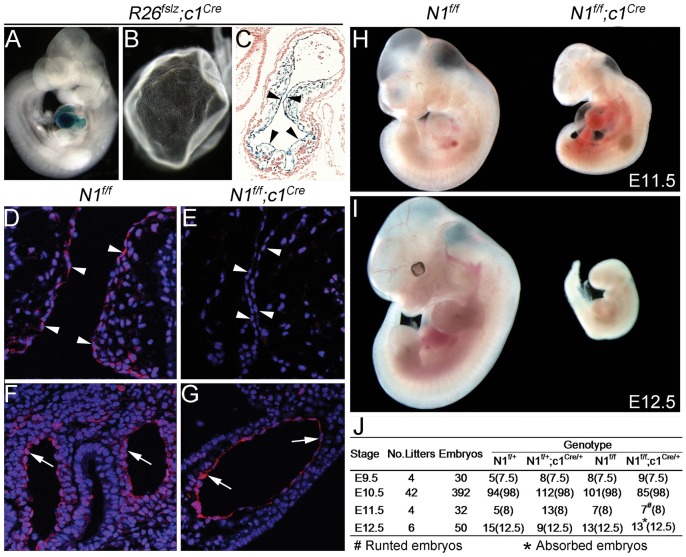
Loss of Notch1 in the endocardium is embryonic lethal. **A** and **B,** wholemount X-gal staining of the E10.5 *R26^fslz^;c1^Cre^* embryo and yolk sac showing that the Cre recombinase-mediated LacZ expression (blue) was restricted to the heart (**A**) and not present in the yolk sac (**B**). **C,** sections of the X-gal stained embryos showing that LacZ expression was localized in the endocardium (arrowheads) and endocardial-derived cushion mesenchymal cells at the atrioventricular canal (AVC). **D–G,** Immunofluorescence showing that Notch1 protein is present in the AVC endocardium (**D**, arrowheads) and the pharyngeal vascular endothelium of *N1* ***^f/f^*** embryo (**F**, arrows), but not in the AVC endocardium of *N1* ***^f/f^*** *;c1* ***^Cre^*** embryo (**E**, arrowheads). Note that Notch1 protein remains in the pharyngeal vascular endothelium of *N1* ***^f/f^*** *;c1* ***^Cre^*** embryo (**G**, arrows). **H** and **I,** Wholemount views showing that E11.5 *N1* ***^f/f^*** *;c1* ***^Cre^*** embryos were runted and had dilated pericardial sac (**H**) and E12.5, *N1* ***^f/f^*** *;c1* ***^Cre^*** embryos were absorbed (**I**). **J,** summarizing the total number of embryos analyzed at different stages, indicating that *N1* ***^f/f^*** *;c1* ***^Cre^*** embryos died between E11.5 and E12.5. The expected number of embryos at different stages is indicated in the parentheses.

To further characterize the role of Notch signaling in the endocardium for heart development, we deleted *Notch1* or *Jagged1* in the endocardium during early mouse embryogenesis using the *Nfatc1-Cre* mouse (*c1^Cre^*) [21], a knockin Cre at the *Nfatc1* locus which codes an endocardial-specific gene [28], [32], [33], [34], [35]. As shown in Figure 1A and 1C, *c1^Cre^* activated *lacZ* expression specifically in the endocardium and its mesenchymal derivatives of E10.5 Rosa26 floxed-stop-lacZ (*R26^fslz^*) embryos. However, the *lacZ* expression was not found in the yolk sac (Figure 1B). We crossed *c1^Cre^* with floxed *Notch1* mice (*N1^f/f^*) to generate endocardial *Notch1* knockout embryos (*N1^f/f^;c1^Cre^*, hereafter) and confirmed by immunofluorescent staining that Notch1 protein in endocardium (but not vascular endothelium) was abolished at E10.5 (Figure 1D to 1G). All *N1^f/f^;c1^Cre^* embryos died between E11.5 and E12.5 with severe pericardial effusion (Figure 1H to 1J), suggesting insufficient heart function. Of note, the timing of embryonic death in our study is different from that in previous studies of germline or pan-endothelial deletion of *Notch1*, which cause embryonic death before E10.5 [17], [18].

**Figure 2:**
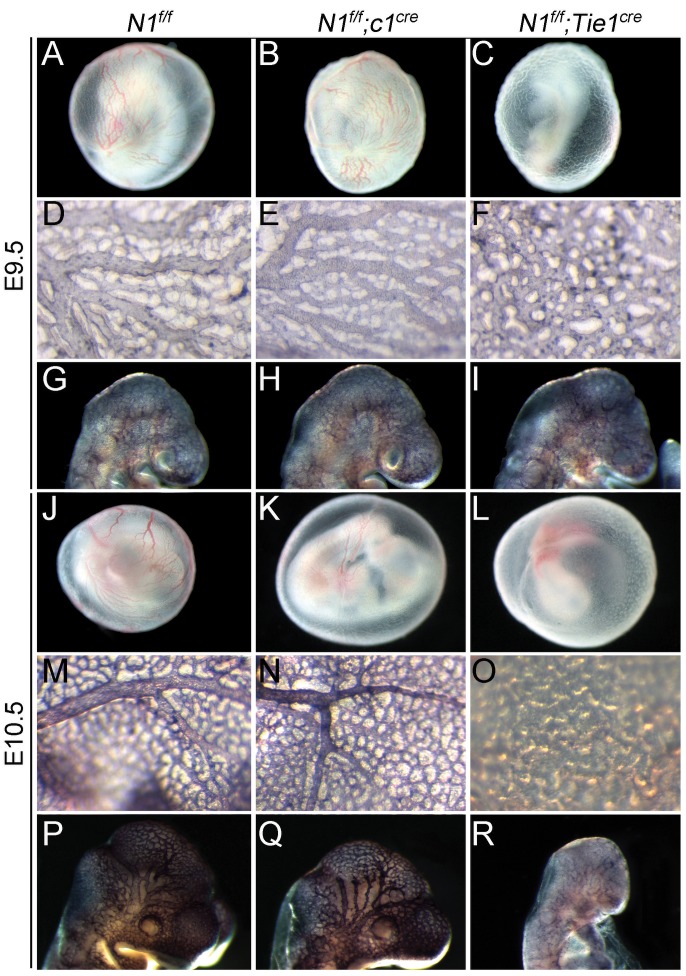
Endocardial-specific deletion of *Notch1* does not affect early vascular formation. **A**–**C,** wholemount view showing the yolk sac vessels in E9.5 *N1^f/f^* (**A**) and *N1^f/f^;c1^Cre^* (**B**) embryos; they are not present in the *N1^f/f^;Tie1^Cre^* embryos (**C**). **D–I,** Pecam1 staining showing mature vessels in the yolk sac and head of *N1^f/f^* (**D** and **G**) and *N1^f/f^;c1^Cre^* embryos (**E** and **H**); they are not formed in the *N1^f/f^;Tie1^Cre^* embryos (**F** and **I**). **J–L,** wholemount view showing mature yolk sac vessels in E10.5 *N1^f/f^* (**J**) and *N1^f/f^;c1^Cre^* (**K**), but not in *N1^f/f^;Tie1^Cre^* embryos (**L**). **M–R,** Pecam1 staining showing mature vascular networks in the yolk sac and head of E10.5 *N1^f/f^* (**M** and **P**) and *N1^f/f^;c1^Cre^* (**N** and **Q**) but not *N1^f/f^;Tie1^Cre^* (**O** and **R**) embryos.

We then compared control *N1^f/f^, N1^f/f^;c1^Cre^*, and pan-endothelial *Notch1* null embryos (*N1^f/f^;Tie1^Cre^*) (Figure 2). *N1^f/f^;c1^Cre^* embryos developed to E9.5–E10.5 with global appearance comparable to control *N1^f/f^* embryos (Figure 2A, 2J vs. 2B and 2K). However, *N1^f/f^;Tie1^Cre^* embryos were runted (Figure 2C and 2L). Pecam1 immunostaining showed normal vessels in the yolk sac (Figure 2D and 2E) and head (Figure 2G and 2H) of E9.5 *N1^f/f^* and *N1^f/f^;c1^Cre^* embryos, but not in *N1^f/f^;Tie1^Cre^* embryos (Figure 2F and 2I). By E10.5 we noticed small differences in gross morphology of yolk sac and head vessels between *N1^f/f^* embryos (Figure 2M and 2P) and *N1^f/f^;c1^Cre^* embryos (Figure 2N and 2Q), but the patterning of vasculature appeared normal, suggesting a potential defect in vascular development secondary to insufficient cardiac function. In contrast, early vascular development in *N1^f/f^;Tie1^Cre^* embryos at this stage was completely arrested (Figure 2O and 2R), as described before [18]. In addition, we generated endocardial *Jagged1* knockout (*J1^f/f^;c1^Cre^*) mice using the *c1^Cre^* and floxed *Jagged1* mice (*J1^f/f^*) and confirmed its endocardial deletion by immunofluorescence (Figure S1). This deletion resulted in similar defects in vascular development (data not shown). Our results demonstrate that the Jagged1-Notch1 signaling in the endocardium is essential for embryonic survival, likely through its primary functions in the regulation of heart development [11], [12], [16], [36], [37], [38].

### Disruption of Endocardial Jagged1-Notch1 Signaling Blocks EMT and Endocardial Cushion Formation

**Figure 3:**
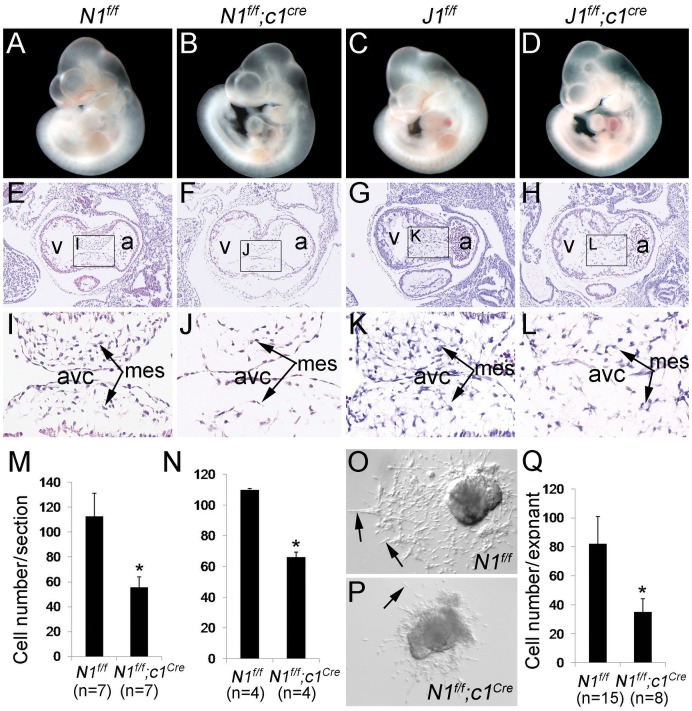
Disruption of endocardial Jagged1-Notch1 signaling blocks EMT and formation of endocardial cushions. **A**–**D**, wholemount views showing that at E10.5 the gross morphology was comparable between *N1^f/f^* (**A**) and*N1^f/f^;c1^Cre^* (**B**) embryos. Similar results were observed between *J1^f/f^* (**C**) and *J1^f/f^;c1^Cre^* (**D**) embryos. **E–L,** H&E stained sections through the atrioventricular canal (avc) region of E10.5 embryos showing dense mesenchymal cells (arrows) in the cushion of *N1^f/f^* (**E** and **I**) or *J1^f/f^* (**G** and **K**) hearts, but fewer mesenchymal cells in the same region of *N1^f/f^;c1^Cre^* (**F** and **J**) or *J1^f/f^;c1^Cre^* (**H** and **L**) hearts. **M** and **N,** quantitative analysis of the number of mesenchymal cells in the cushion of *N1^f/f^;c1^Cre^* (**M**) or *J1^f/f^;c1^Cre^* (**N**) hearts. *p<0.001. **O–Q,** *in vitro* collagen gel EMT assay showing that while ∼80 endocardial cells (arrows) migrated away from each *N1^f/f^* explant (**O** and **Q**), fewer (25/explant) cells underwent this process in cultured *N1^f/f^;c1^Cre^* explants (**P** and **Q**). *p<0.001.

We then examined E10.5 *N1^f/f^;c1^Cre^* or *J1^f/f^;c1^Cre^* embryos and found that they appeared normal comparing to their *N1^f/f^* or *J1^f/f^* littermates (Figure 3A to 3D). However, on cross sections, we noted that while mesenchymal cells derived from cushion endocardial cells via EMT had populated endocardial cushions at the AVC of *N1^f/f^* (Figure 3E and 3I) and *J1^f/f^* embryos (Figure 3G and 3K), much fewer mesenchymal cells were present in the cushions of *N1^f/f^;c1^Cre^* (Figure 3F and 3J) and *J1^f/f^;c1^Cre^* embryos (Figure 3H and 3L). Quantitative analysis showed significant reduction in the number of mesenchymal cells in AVC cushions of *N1^f/f^;c1^Cre^* and *J1^f/f^;c1^Cre^* embryos (Figure 3M and 3N). We also observed the same phenotype in the outflow tract (OFT) cushions of *N1^f/f^;c1^Cre^* (Figure S2, A, B vs. C, D) and *J1^f/f^;c1^Cre^* embryos (Figure S2, E, F vs. G, H). In addition to EMT and cushion defects, *N1^f/f^;c1^Cre^* and *J1^f/f^;c1^Cre^* embryos had poor trabeculation (data not shown).

To identify the cellular defects that contributed to the hypocellular cushions, we studied cell proliferation by BrdU labeling and apoptosis by Caspase3 staining. The results indicated that the numbers of proliferating cells in the endocardium or cushion mesenchyme were comparable between E10.5 *N1^f/f^* and *N1^f/f^;c1^Cre^* embryos (Figure S3). Additionally, there were no detectable apoptotic cells in the endocardial cushions of E10.5 *N1^f/f^* and *N1^f/f^;c1^Cre^* embryos (data not shown). We then studied EMT process by fate mapping cushion endocardial cells in E10.5 control (*N1^f/+^;R26^fslz^;c1^Cre^*) and endocardial *Notch1* knockout (*N1^f/f^;R26^fslz^;c1^Cre^*) embryos. X-gal staining showed that LacZ-positive mesenchymal descendants of endocardial cells populated the AVC cushions of *N1^f/+^;R26^fslz^;c1^Cre^* embryos (Figure S4, A); in contrast, the number of LacZ-positive mesenchymal cells was dramatically reduced in the AVC cushions of *N1^f/f^;R26^fslz^;c1^Cre^* embryos (Figure S4, B). The same result was found at OFT cushions (Figure S4, C and D). We subsequently examined EMT by culturing AVC of E9.5 hearts on collagen gels. We found that while endocardial cells migrated away from *N1^f/f^* explants and became elongated mesenchymal cells that invaded the gel (Figure 3O and 3Q), fewer endocardial cells underwent EMT in *N1^f/f^;c1^Cre^* AVC explants (Figure 3P and 3Q). Our findings are consistent with previous observations made in the standard knockout [11] or pan-endothelium knockout [12] and confirm that endocardial Jagged1-Notch1 signaling is required for EMT.

### Endocardial Jagged1-Notch1 Signaling Regulates Expression of Endocardial *Wnt4* and Myocardial *Bmp2*

**Figure 4:**
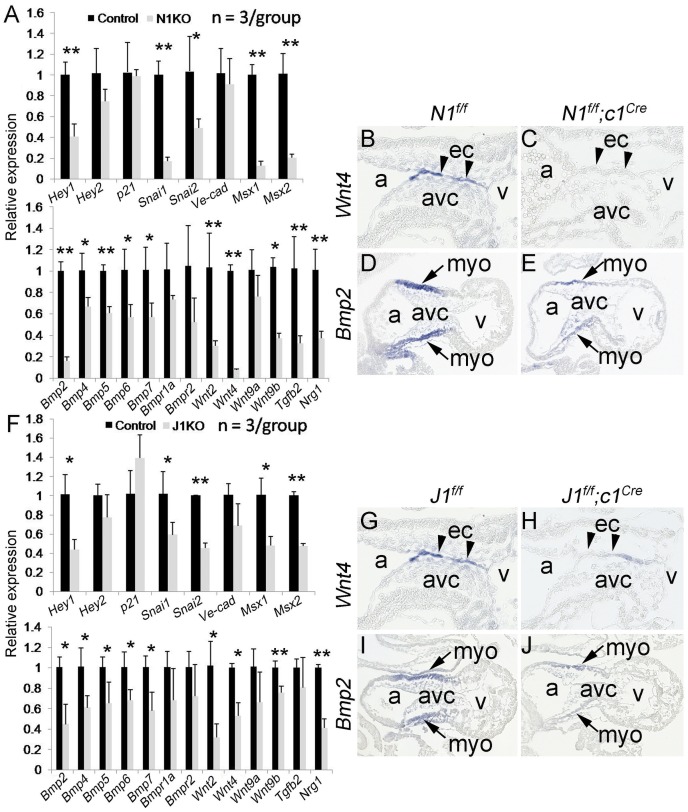
Endocardial Jagged1-Notch1 signaling regulates expression of endocardial *Wnt4* and myocardial *Bmp2*. **A**, qPCR analysis of EMT gene expression in the atrioventricular canal (avc) from E10.5 *N1^f/f^* or *N1^f/f^;c1^Cre^* hearts. Each cDNA sample was prepared from five avc tissues and three samples of each group were used for qPCR. Gene expression was normalized to *Gapdh*. *p<0.05; **p<0.01. **B–E,** RNA *in situ* hybridization showing endocardial *Wnt4* (**B**, ec, arrowheads) and myocardial *Bmp2* expression (**D**, myo, arrows) in E10.5 *N1^f/f^* hearts. Their expression is dramatically reduced in *N1^f/f^;c1^Cre^* hearts (**C** and **E**). **F,** qPCR analysis of EMT gene expression in the AVC cushions of E10.5 *J1^f/f^* or *J1^f/f^;c1^Cre^* hearts. **G–J,** RNA *in situ* hybridization showing that *Wnt4* and *Bmp2* expression is downregulated in *J1^f/f^;c1^Cre^* hearts. a, atrium and v, ventricle.

To identify the factors that mediate endocardial Jagged1-Notch1 signaling for EMT, we examined expression of Notch targets and genes known to be involved in EMT by qPCR. The results showed that expression of *Hey1*, a major nuclear factor mediating canonical Notch signaling [39], was significantly reduced in AVC cushions of E10.5 *N1^f/f^;c1^Cre^* hearts, whereas expression of *p21*, a non-canonical Notch effector [39], was not affected (Figure 4A). Expression of *Snai1*, *Snai2*, *Msx1*, and *Msx2*, transcription factors involved in EMT [11], [40], [41] was also downregulated in *N1^f/f^;c1^Cre^* hearts. However, expression of *Ve-cad* was unchanged in AVC cushions of E10.5 *N1^f/f^;c1^Cre^* hearts. Interestingly, we found that expression of *Wnt2*, *Wnt4*, *Wnt9b*, *Bmp2*, *Bmp4*, *Bmp5*, *Bmp6*, and *Bmp7* was significantly decreased in the *N1^f/f^;c1^Cre^* AVC cushions (Figure 4A). In addition, expression of *Tgfb2* and *Nrg1* was decreased in the AVC cushions of E10.5 *N1^f/f^;c1^Cre^* hearts. Using RNA *in situ* hybridization, we further examined expression of *Wnt4* and *Bmp2*, which are specific for AVC endocardium and myocardium, respectively. The results showed that their expression was abolished in the E10.5 *N1^f/f^;c1^Cre^* hearts (Figure 4B to 4E). Similar changes in gene expression were observed in E10.5 *J1^f/f^;c1^Cre^* hearts using qPCR (Figure 4F) and RNA *in situ* hybridization (Figure 4G to 4J). These results indicate that endocardial Jagged1-Notch1 signaling regulates endocardial Wnt and myocardial Bmp activities to ensure proper EMT at the valve ontogenic site. They also suggest that endocardially produced Wnt ligands may be responsible for Bmp2 expression in the adjacent myocardium.

### Endocardial Wnt Ligands Regulate Myocardial *Bmp2* Expression

**Figure 5:**
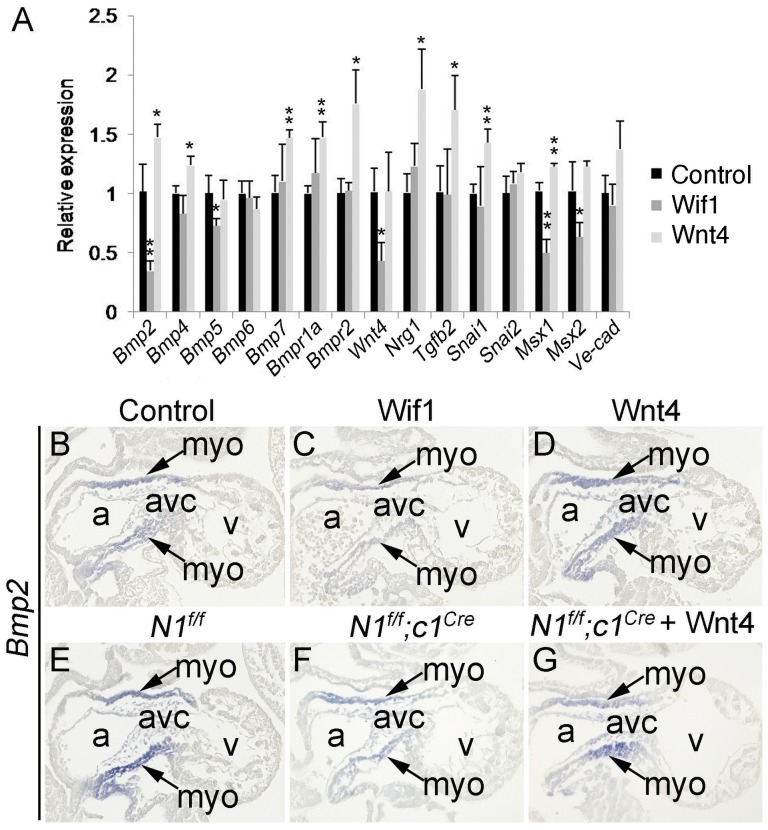
*Bmp2* expression is regulated by Wnt signaling. **A**, qPCR analysis showing that *Bmp2* expression was inhibited by Wif1 and induced by Wnt4. E9.5 embryos were cultured in the control media or media with Wnt inhibitor Wif1 or recombinant mouse Wnt4. After 24-hour culture, each RNA sample was prepared from AVC tissues of 5 hearts for each treatment. The data from three independent samples for each group were used for statistical calculation. *p<0.05 and **p<0.01. **B–D,** RNA *in situ* hybridization analysis showing *Bmp2* expression (indicated by arrows) in cultured wild type embryos under indicated condition; cushion myocardial *Bmp2* expression was inhibited by Wif1 (**C**) and induced by Wnt4 (**D**). **E–G,** RNA *in situ* hybridization showing *Bmp2* expression in cultured *N1^f/f^* (**E**), *N1^f/f^;c1^Cre^* embryos (**F**), and *N1^f/f^;c1^Cre^* embryo treated with Wnt4 (**G**). The data indicated that *Bmp2* expression was reduced in the *N1^f/f^;c1^Cre^* embryo when compared to the *N1^f/f^* embryo. However, Wnt4 treatment restored its expression in the *N1^f/f^;c1^Cre^* embryo (**G**). a, atrium and v, ventricle.

To determine whether expression of *Bmp2* and other genes involved in the EMT was regulated by Wnt ligands, we cultured E9.5 embryos for one day with Wnt antagonist Wif1 [42] or recombinant mouse Wnt4 protein and examined their effect on the expression of genes listed in Figure 4A using qPCR. The results showed that among the genes examined, expression of *Bmp2* and *Msx1* was significantly decreased after Wif1 treatment, and conversely, significantly increased after Wnt4 treatment (Figure 5A). We further confirmed the Wnt-dependent expression of *Bmp2* in the AVC myocardium using RNA *in situ* hybridization (Figure 5B to 5D). Moreover, Wnt4 treatment recovered *Bmp2* expression in the AVC myocardium of cultured E9.5 *N1^f/f^;c1^Cre^* embryos (Figure 5E to 5G). These results demonstrate that cushion myocardial *Bmp2* expression is regulated by Wnt signals from the adjacent AVC endocardium. This most likely occurs through the cushion endocardial-specific Wnt ligand Wnt4, whose expression is dependent upon endocardial Jagged1-Notch1 signaling.

### Endocardial to Myocardial Notch-Wnt-Bmp Signaling Axis Regulates EMT

**Figure 6:**
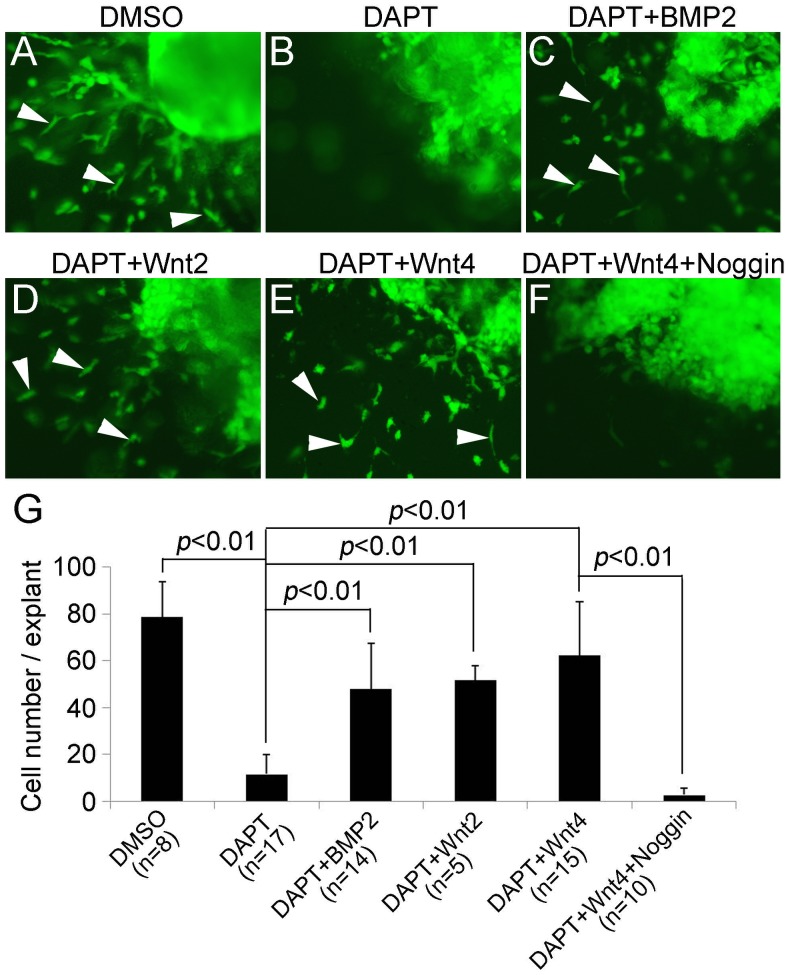
Wnt4 and Bmp2 act downstream of Notch to regulate EMT. **A**, showing *in vitro* collagen gel EMT assay analysis of AVC explants from E9.5 *R26^fsGFP^;c1^Cre^* embryos in which the endocardial cells were labeled by GFP (indicated by arrowheads). **B**, showing that Notch inhibitor DAPT blocked EMT by endocardial cells. **C–E**, Bmp2, Wnt2, or Wnt4 treatment rescued EMT defect caused by DAPT. **F**, showing that Bmp inhibitor Noggin abolished the Wnt4 rescuing. **G,** showing quantitative analysis of the number of transformed mesenchymal cells under each condition. The number of explants analyzed is indicated in parentheses.

To further determine whether Wnt-dependent *Bmp2* expression functions directly downstream of Notch signaling to mediate EMT, we performed rescue experiments using the collagen gel EMT assay. We cultured AVC explants isolated from E9.5 *R26^fsGFP^;c1^Cre^* embryos, in which the cushion endocardial cells and their mesenchymal descendants were marked by GFP expression, allowing direct visualization of migration and invasion by endocardial cells (Figure 6A). While explants treated with DAPT to block Notch activities abolished EMT by the endocardial cells (Figure 6B and 6G), Wnt2, Wnt4, or BMP2 treatment was able to rescue EMT defect caused by inhibition of Notch signaling (Figure 6C, 6D or 6E, respectively). Of particular note, blocking Bmp activities with Noggin attenuated rescue of impaired EMT by Wnt4 (Figure 6E vs. 6F). Together, these results establish that endocardial Notch-Wnt signaling promotes EMT through myocardial Bmp2 at the valve ontogenic region.

## Discussion

Previous studies have shown that Notch, Wnt, and Bmp signals in the endocardium and myocardium of the valve ontogenic site regulate EMT and early heart valve formation [1], [11], [12], [16], [36], [37], [38], [43], [44]. In addition, interactions between Notch and BMP signaling have been found in the developing endocardial cushions [15], [16]. This study further addresses how endocardial and myocardial signals coordinate to direct EMT. We used genetic knockouts, EMT assays, and rescue experiments to uncover a signaling cascade from endocardial Notch-Wnt to myocardial Bmp that directs EMT to form the primitive heart valves.

We show that endocardial-specific *Notch1* or *Jagged1* knockout embryos are embryonic lethal and they die around E11.5 with pericardial effusion (Figure 1). This finding is of significance as endocardial *Notch1* or *Jagged1* null embryos develop normal vascular networks in the yolk sac and embryo at E9.5–10.5, whereas embryos with pan-endothelial loss of *Notch1* or *Jagged1* die around E9.5–10.5 with severely defective vascular angiogenesis (Figure 2, data not shown) as shown previously [18], [19]. Thus, our mouse models allowed us localize the primary role of endocardial Jagged1-Notch1 signaling in EMT and early heart valve development (Figure 3), which has been described in previous studies [11], [12], [16], [38].

At the molecular level, our study shows that endocardial *Notch1* or *Jagged1* knockout embryos have reduced expression of key genes involved in the EMT. This includes transcription factors *Hey1*, *Snai1*, *Snai2*, *Msx1*, and *Msx2*. *Snai1* and *Snai2* are known to suppress expression of *VE-cad*, which is necessary for initiation of EMT [36], [41], [45], [46]. However, we did not observe a significant change in the level of *VE-cad* expression in our endocardial *Notch1* or *Jagged1* knockout hearts. This discrepancy might be due to the difference between the embryonic stages analyzed in the previous and our studies.

Perhaps the most interesting observation in this study is that Wnt and Bmp, two critical signaling pathways that have been shown to regulate EMT and heart valve formation [1], are dramatically attenuated in AVC cushions of endocardial *Notch1* or *Jagged1* knockout hearts. Specifically, expression of Wnt ligands (*Wnt2*, *Wnt4*, *Wnt9b*), and Bmp ligands (*Bmp2*, *Bmp4*, *Bmp5*, *Bmp6*, and *Bmp7*) is reduced in the cushions. Of these genes, *Wnt4* and *Bmp2* are predominantly expressed by AVC endocardium and myocardium, respectively, during EMT and early valve formation. This observation suggests that endocardially derived *Wnt4* is downstream of Jagged1-Notch1 signaling to mediate myocardial *Bmp2* expression. Indeed, further pharmacological experiments using the whole embryo culture show that *Bmp2* expression in the AVC myocardium is dependent on Wnt activities, as the Wnt inhibitor Wif1 suppresses Bmp2 expression. On the other hand, Wnt4 is sufficient to induce myocardial *Bmp2* expression (Figure 4). Moreover, the rescue experiments demonstrate that Wnt4 can restore *Bmp2* expression in the AVC myocardium of endocardial *Notch1* knockout embryos (Figure 5). Together these results suggest that endocardial Notch-Wnt signaling regulates cushion myocardial *Bmp2* expression.

We do not know how endocardial Notch signaling regulates expression of Wnt ligands in the endocardium. Further studies are needed to determine whether canonic or non-canonic Notch signaling or both regulate expression of Wnt. Similarly, how endocardial Wnt regulates myocardial Bmp2 expression needs to be investigated in the future. In addition, previous studies have shown that the levels of cushion myocardial *Bmp2* expression are not changed in the *Rbpj* germline knockout embryos [16]. However, our study indicates that endocardial Notch signaling is required for *Bmp2* expression in the AVC myocardium. This discrepancy might be due to the difference in the embryonic stages analyzed in the previous and our studies. Alternatively, *Rbpj* mutant affects mainly the canonic Notch signaling, whereas endocardial *Notch1* deletion impairs both canonic and non-canonic signaling.

It is well known that signals from the AVC myocardium induce EMT by the endocardial cells [4], [6], [7], [8]. For instance, myocardial Bmp2 regulates endocardial *Notch1* expression and is required for EMT [15], [16]. Our findings indicate that endocardial Notch signaling regulates *Bmp2* expression in the AVC myocardium. We also show that multiple Wnt ligands are downregulated in the endocardial *Notch1* or *Jagged1* knockout hearts (Figure 4) and either Wnt2 or Wnt4 can rescue the impaired EMT resulting from Notch inhibition (Figure 6). It is worth to mention that *Wnt4* knockout mice survive to birth [47], [48], whereas *Wnt2*-null AVC explants exhibit poor EMT [49]. Loss of the common nuclear effector of Wnt signaling, beta-catenin, in the endocardium, results in severely defective EMT and hypocelluar endocardial cushions [13]. These observations suggest that the redundant expression of Wnt ligands in the AVC endocardium [50], also found in our study (Figure 4), compensate each other’s function.

**Figure 7:**
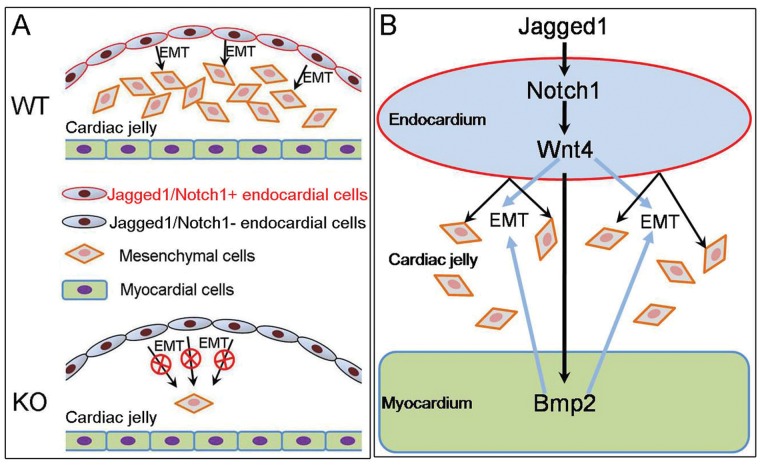
Working model shows Notch-Wnt-Bmp signaling axis that regulates EMT and early valve formation. **A**, Schematic showing the cardiac phenotypes found in the endocardial *Jagged1* or *Notch1* knockout embryos. During E9.5-E10.5, cushion endocardial cells undergo EMT and form endocardial cushions at the atrioventricular canal and outflow tract of the wild-type (WT) embryos. This process is disrupted in the endocardial *Jagged1* (*J1^f/f^;c1^Cre^*) or *Notch1* (*N1^f/f^;c1^Cre^*) knockout (KO) embryos, which results in hypocellular endocardial cushions. **B,** Endocardial Jagged1-mediated Notch1 activation induces expression of Wnt4, which subsequently upregulates expression of Bmp2 in the adjacent myocardium. Myocardial Bmp2 then acts on endocardial cells to promote EMT. This Notch-Wnt-Bmp signaling axis promotes EMT during heart valve development.

Together with previous studies [15], [16], our study suggests a reciprocal Notch-Bmp signaling between the endocardium and myocardium at the valve ontogenic site. This endocardial-myocardial signaling begins with endocardial Jagged1-Notch1 signaling; it regulates expression of endocardial Wnt4 or other Wnt ligands, which induces myocardial Bmp2 expression; myocardial Bmp2 then promotes endocardial cells to undergo EMT [15], [51] (Figure 7). This study thus improves the current understanding of EMT in early heart valve development and may provide new insights into the genesis of congenital valve defects.

## Supporting Information

Figure S1 **Endocardial deletion of** ***Jagged1*** **by** ***C1^Cre^*** **.** Immunofluorescence showing Jagged1 protein in the cushion endocardial cells (arrows) in E10.5 *J1^f/f^* hearts (**A**). In contrast, the level of Jagged1 protein in the endocardium was greatly reduced in the *J1^f/f^;c1^Cre^* hearts (**B**).
(TIF)

Figure S2 **Hypocellular cushions at the outflow tract region in endocardial** ***Notch1*** **or** ***Jagged1*** **null embryos.** Histological analysis of E10.5 embryo sections through the outflow tract (OFT) region showing that endocardial *Notch1* knockout (*N1^f/f^;c1^Cre^*) (**C** and **D**) or *Jagged1* knockout (*J1^f/f^;c1^Cre^*) (**G** and **H**) hearts have hypocellular OFT cushions compared to control *N1^f/f^* (**A** and **B**) or *J1^f/f^* (**E** and **F**).
(TIF)

Figure S3 **Endocardial specific disruption of** ***Notch1*** **does not affect cell proliferation in the endocardial cushions. A–C,** BrdU incorporation and immunostaining showing that the percentage of proliferating cells in the endocardium (arrowheads) or cushion mesenchyme (arrows) is comparable at the atrioventricular canal (avc) between the control (*N1^f/f^*) and endocardial *Notch1* knockout (*N1^f/f^;c1^Cre^*) embryos. Serial sections throughout the avc of each embryo were used for cell counting and data from three embryos from each group were analyzed for statistical significance.
(TIF)

Figure S4 **Lineage tracing of endocardial cells in endocardial** ***Notch1*** **knockout hearts. A–D,** photos of X-gal stained sections through the endocardial cushions of E10.5 hearts showing that the descendants of endocardial cells contribute a dense cushion mesenchyme to the atrioventricular canal (avc, **A**) and outflow tract (oft, **C**) cushions of the *N1^f/+^;R26^fslz^;c1^Cre^* embryos. Such contribution is reduced in the *N1^f/f^;R26^fslz^;c1^Cre^* embryos (**B** and **D**).
(TIF)

Table S1 List of primers used in qPCR.
(DOCX)
