# Supplementary figures and images for: Endocardial to Myocardial Notch-Wnt-Bmp Axis Regulates Early Heart Valve Development
Source: PLoS One. 2013 Apr 1;8(4):e60244. doi: 10.1371/journal.pone.0060244 (PMC3613384; doi:10.1371/journal.pone.0060244)

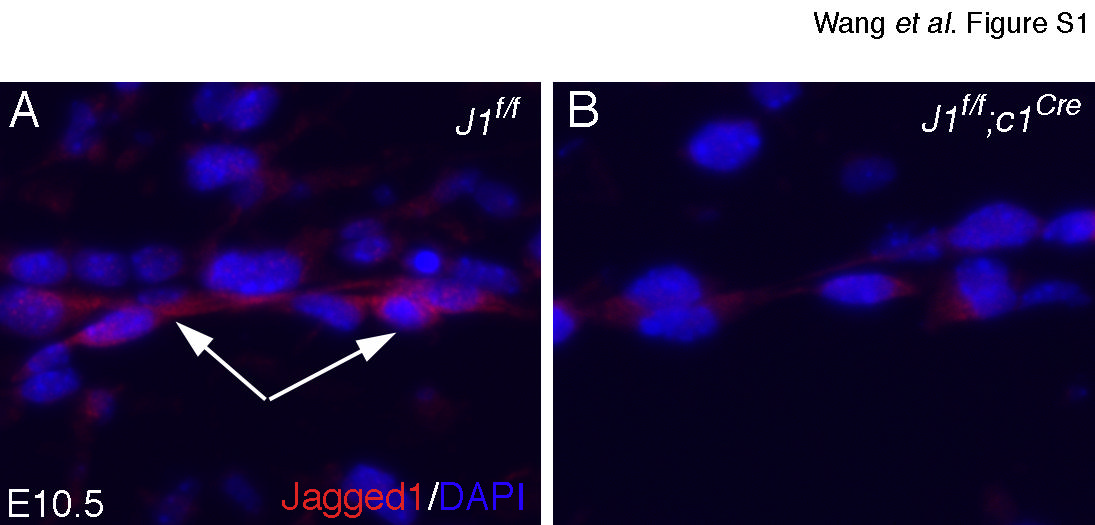

Supplement: Figure S1 — Endocardial deletion of Jagged1 by C1Cre . Immunofluorescence showing Jagged1 protein in the cushion endocardial cells (arrows) in E10.5 J1f/f hearts (A). In contrast, the level of Jagged1 protein in the endocardium was greatly reduced in the J1f/f;c1Cre hearts (B). (TIF) [file pone.0060244.s001.tif]

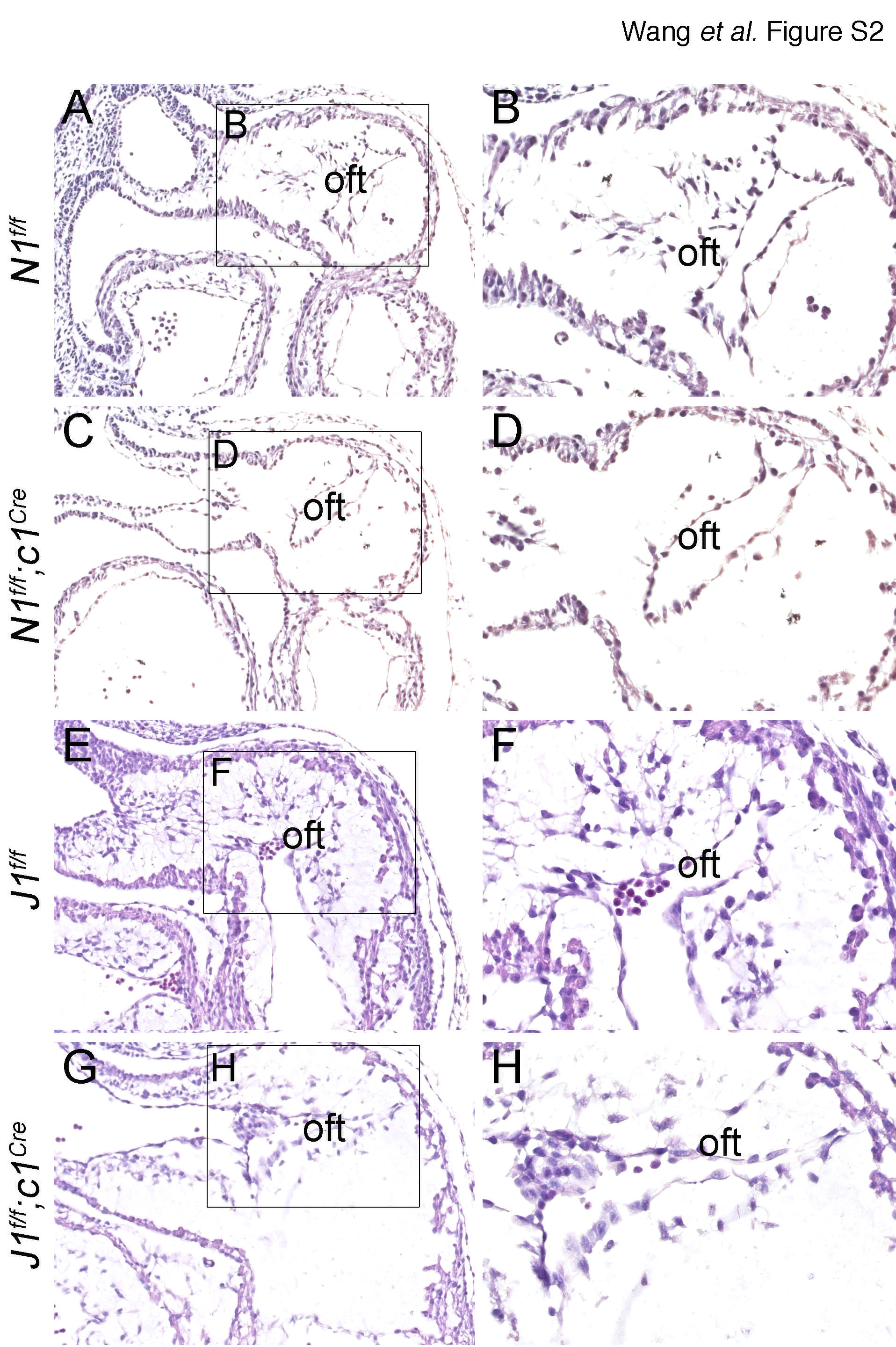

Supplement: Figure S2 — Hypocellular cushions at the outflow tract region in endocardial Notch1 or Jagged1 null embryos. Histological analysis of E10.5 embryo sections through the outflow tract (OFT) region showing that endocardial Notch1 knockout (N1f/f;c1Cre) (C and D) or Jagged1 knockout (J1f/f;c1Cre) (G and H) hearts have hypocellular OFT cushions compared to control N1f/f (A and B) or J1f/f (E and F). (TIF) [file pone.0060244.s002.tif]

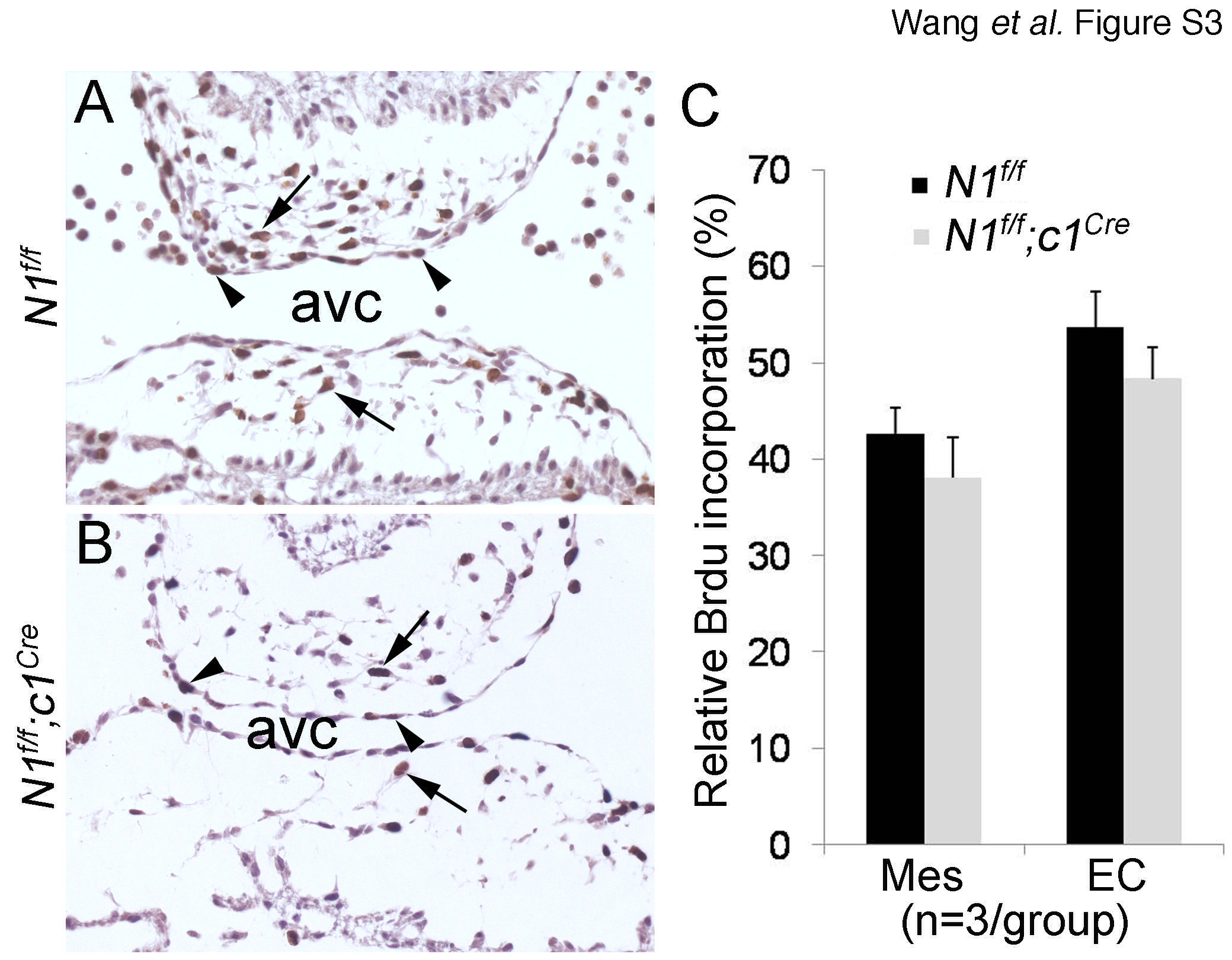

Supplement: Figure S3 — Endocardial specific disruption of Notch1 does not affect cell proliferation in the endocardial cushions. A–C, BrdU incorporation and immunostaining showing that the percentage of proliferating cells in the endocardium (arrowheads) or cushion mesenchyme (arrows) is comparable at the atrioventricular canal (avc) between the control (N1f/f) and endocardial Notch1 knockout (N1f/f;c1Cre) embryos. Serial sections throughout the avc of each embryo were used for cell counting and data from three embryos from each group were analyzed for statistical significance. (TIF) [file pone.0060244.s003.tif]

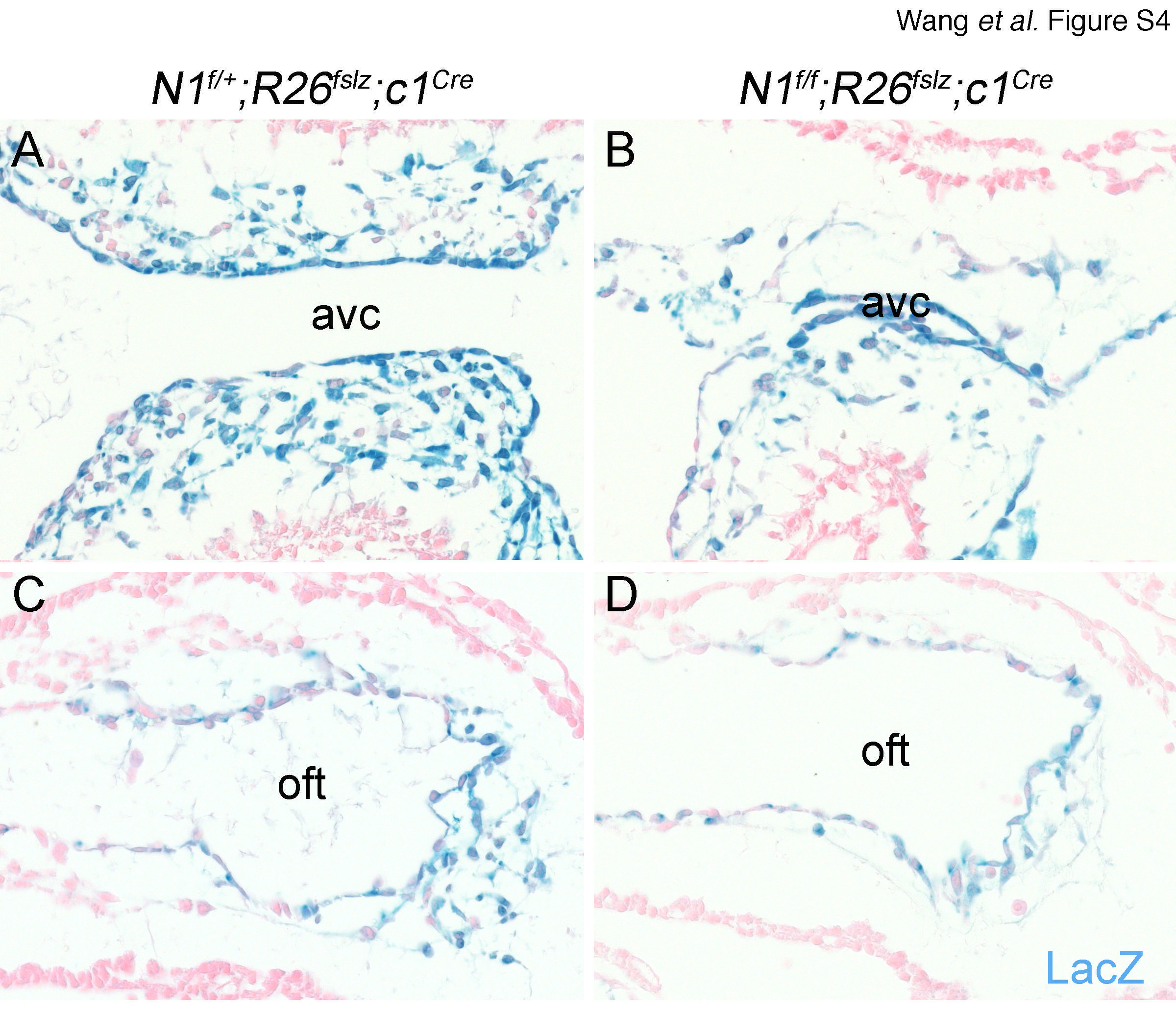

Supplement: Figure S4 — Lineage tracing of endocardial cells in endocardial Notch1 knockout hearts. A–D, photos of X-gal stained sections through the endocardial cushions of E10.5 hearts showing that the descendants of endocardial cells contribute a dense cushion mesenchyme to the atrioventricular canal (avc, A) and outflow tract (oft, C) cushions of the N1f/+;R26fslz;c1Cre embryos. Such contribution is reduced in the N1f/f;R26fslz;c1Cre embryos (B and D). (TIF) [file pone.0060244.s004.tif]
