# Supplementary material for: Endocardial to Myocardial Notch-Wnt-Bmp Axis Regulates Early Heart Valve Development
Source: PLoS One. 2013 Apr 1;8(4):e60244. doi: 10.1371/journal.pone.0060244 (PMC3613384; doi:10.1371/journal.pone.0060244)
Supplement: Table S1 — List of primers used in qPCR. (DOCX) [file pone.0060244.s005.docx]

Wang *et al.* Table S1

| **Gene** | **Primer sequence** | | **Product size** | |
| --- | --- | --- | --- | --- |
| *Bmp2* | | Forward: 5'-TGTGGGCCCTCATAAAGAAGCAGA-3' | | 165 bp |
|  |  | Reverse: 5'-AGATCCCTGCTTCTCAAAGGCACT-3' | |  |
| *Bmp4* | | Forward: 5'-TGGCTCCCAAGAATCATGGACTGT-3' | | 84 bp |
|  |  | Reverse: 5'-AGATCCCTGCTTCTCAAAGGCACT-3' | |  |
| *Bmp5* | | Forward: 5'-GGATGGCCGCAGCATCAATGTAAA-3' | | 85 bp |
|  |  | Reverse: 5'-AGATCCCTGCTTCTCAAAGGCACT-3' | |  |
| *Bmp6* | | Forward: 5'-AGAAGGGCACTCTTTCAGGTTCCA-3' | | 140 bp |
|  |  | Reverse: 5'-AGATCCCTGCTTCTCAAAGGCACT-3' | |  |
| *Bmp7* | | Forward: 5'-ACCGCAGCCGAATTCAGGATCTAT-3' | | 175 bp |
|  |  | Reverse: 5'-AGATCCCTGCTTCTCAAAGGCACT-3' | |  |
| *Bmpr1a* | | Forward: 5'-AATGCAAGGATTCACCGAAAGCCC-3' | | 160 bp |
|  |  | Reverse: 5'-AGATCCCTGCTTCTCAAAGGCACT-3' | |  |
| *Bmpr2* | | Forward: 5'-TGGCAGTGAGGTCACTCAAGGAAA-3' | | 160 bp |
|  |  | Reverse: 5'-AGATCCCTGCTTCTCAAAGGCACT-3' | |  |
| *Gapdh* | | Forward: 5'-ACGGCAAATTCAACGGCACAGTCA-3' | | 231 bp |
|  |  | Reverse: 5'-AGATCCCTGCTTCTCAAAGGCACT-3' | |  |
| *Hey1* | | Forward: 5'-GAAACTTGAGTTCGGCGCTGTGTT-3' | | 167 bp |
|  |  | Reverse: 5'-AGATCCCTGCTTCTCAAAGGCACT-3' | |  |
| *Hey2* | | Forward: 5'-AGGCTACTTTGATGCCCATGCTCT-3' | | 80 bp |
|  |  | Reverse: 5'-AGATCCCTGCTTCTCAAAGGCACT-3' | |  |
| *Msx1* | | Forward: 5'-TCCTGGTTGTCGCTTCCTAAACCT-3' | | 151 bp |
|  |  | Reverse: 5'-AGATCCCTGCTTCTCAAAGGCACT-3' | |  |
| *Msx2* | | Forward: 5'-TGAGGAAACACAAGACCAACCGGA-3' | | 96 bp |
|  |  | Reverse: 5'-AGATCCCTGCTTCTCAAAGGCACT-3' | |  |
| *Nrg1* | | Forward: 5'-AAACGACCCAGGAGTATGAGCCAA-3' | | 123 bp |
|  |  | Reverse: 5'-AGATCCCTGCTTCTCAAAGGCACT-3' | |  |
| *p21* | | Forward: 5'-TTGTACAAGGAGCCAGGCCAAGAT-3' | | 119 bp |
|  |  | Reverse: 5'-AGATCCCTGCTTCTCAAAGGCACT-3' | |  |
| *Snia1* | | Forward: 5'-ACAGCTGCTTCGAGCCATAGAACT-3' | | 122 bp |
|  |  | Reverse: 5'-AGATCCCTGCTTCTCAAAGGCACT-3' | |  |
| *Snai2* | | Forward: 5'-ACTACAGCGAACTGGACACACACA-3' | | 171 bp |
|  |  | Reverse: 5'-AGATCCCTGCTTCTCAAAGGCACT-3' | |  |
| *Tgfb2* | | Forward: 5'-TGACCGTGAAGTGGCTGTTGATCT-3' | | 107 bp |
|  |  | Reverse: 5'-AGATCCCTGCTTCTCAAAGGCACT-3' | |  |
| *Ve-cad* | | Forward: 5'-TAGCAAGAGTGCGCTGGAGATTCA-3' | | 89 bp |
|  |  | Reverse: 5'-AGATCCCTGCTTCTCAAAGGCACT-3' | |  |
| *Wnt2* | | Forward: 5'-AACTGCAACACCCTGGACAGAGAT-3' | | 86 bp |
|  |  | Reverse: 5'-AGATCCCTGCTTCTCAAAGGCACT-3' | |  |
| *Wnt4* | | Forward: 5'-ACTGCCCAGGCCAAAGAAATTCAC-3' | | 134 bp |
|  |  | Reverse: 5'-AGATCCCTGCTTCTCAAAGGCACT-3' | |  |
| *Wnt9a* | | Forward: 5'-AGCACTACCAATGAAGCCACTGGA-3' | | 116 bp |
|  |  | Reverse: 5'-AGATCCCTGCTTCTCAAAGGCACT-3' | |  |
| *Wnt9b* | | Forward: 5'-AAGAGAAAGAAAGCCCTGCCTCCT-3' | | 1134 bp |
|  |  | Reverse: 5'-AGATCCCTGCTTCTCAAAGGCACT-3' | |  |
